# A mouse plasma peptide atlas as a resource for disease proteomics

**DOI:** 10.1186/gb-2008-9-6-r93

**Published:** 2008-06-03

**Authors:** Qing Zhang, Rajasree Menon, Eric W Deutsch, Sharon J Pitteri, Vitor M Faca, Hong Wang, Lisa F Newcomb, Ronald A DePinho, Nabeel Bardeesy, Daniela Dinulescu, Kenneth E Hung, Raju Kucherlapati, Tyler Jacks, Katerina Politi, Ruedi Aebersold, Gilbert S Omenn, David J States, Samir M Hanash

**Affiliations:** 1 Fred Hutchinson Cancer Research Center, Seattle, WA 98109, USA; 2 Center for Computational Medicine and Biology, University of Michigan, Ann Arbor, MI 48109, USA; 3 Institute for Systems Biology, Seattle, WA 98103, USA; 4 Dana-Farber Cancer Institute, Harvard Cancer Center, Boston, MA 02115, USA; 5 Center for Applied Cancer Science, Belfer Institute for Innovative Cancer Science, Department of Medical Oncology, Medicine, Genetics, Dana-Farber Cancer Institute, Harvard Medical School, Boston, MA 02114, USA; 6 Massachusetts General Hospital, Harvard Medical School, Boston, MA 02114, USA; 7 Brigham and Women's Hospital, Harvard Medical School, Boston, MA 02115, USA; 8 Center for Cancer Research, Massachusetts Institute of Technology, Cambridge, MA 02139, USA; 9 Memorial Sloan-Kettering Cancer Center, New York, NY 10021, USA; 10 Institute of Molecular Systems Biology, ETH Zurich and Faculty of Science, University of Zurich, 8093 Zurich, Switzerland

## Abstract

We present an in-depth analysis of mouse plasma leading to the development of a publicly available repository composed of 568 liquid chromatography-tandem mass spectrometry runs. A total of 13,779 distinct peptides have been identified with high confidence. The corresponding approximately 3,000 proteins are estimated to span a 7 logarithmic range of abundance in plasma. A major finding from this study is the identification of novel isoforms and transcript variants not previously predicted from genome analysis.

## Abstract 2

A publicly available repository for high-quality peptide and protein data, identified by LC-MS/MS analysis.

## Background

In-depth analysis of the plasma proteome has the potential to yield biomarkers that allow early disease detection, and monitoring of disease progression, regression or recurrence. Mouse models have provided a physiological context in which to explore various aspects of disease pathogenesis and complement the use of cell line models and tissue sampling approaches. Genetically engineered mouse models have been increasingly relied upon to investigate specific molecular alterations associated with human disease. Recent transcriptional profiling and comparative genomic analyses of human and mouse cancers have revealed significant concordance in genomic alterations and expression profiles, thus justifying reliance on mouse models to identify molecular alterations and markers potentially relevant to human cancers and other diseases [1-5].

Genetically engineered mouse models allow investigations of proteomic changes at defined stages of disease development, and exhibit reduced heterogeneity, thus providing greater ease of standardization of blood and tissue sampling and preparation. However, there has been limited comprehensive analysis to date of the mouse plasma proteome. Studies of disease related plasma protein alterations in the mouse will benefit from a publicly available plasma proteome database that assembles high quality queryable data and that informs about the extent of protein variation encompassed within the mouse proteome.

Previous studies of mouse proteomes include a large-scale study of mouse liver tissue that identified 3,244 proteins [6]. A comparative proteomics study of tumor and normal mammary tissue from a conditional HER2/Neu-driven mouse model of breast cancer identified changes in tissue proteins leading to the identification of up-regulated fibulin-2 and osteopontin in mouse plasma [7]. A study of the plasma proteome in a mouse intestinal tumor model identified a protein subset that distinguished tumor bearing mice from controls [8].

We have implemented a proteomic strategy that allows in-depth analysis of the plasma proteome. We have applied this to protein digests of fractionated mouse plasma reference specimens to determine protein and peptide constituents of mouse plasma and have built a related data repository. A large number of novel transcript variants for mouse plasma proteins have been identified. The data are publicly available at the PeptideAtlas site [9], which can be viewed and searched. The raw data as well as the search results are also available for download from the 'Data Repository' page of the same site.

## Results

### Identification of 13,779 distinct peptides in mouse plasma

Four mouse reference plasma pools were each subjected to extensive fractionation and separate liquid chromatography-tandem mass spectrometry (LC-MS/MS) analysis of digests from individual fractions. The combined four experiments yielded 800,507 spectra with a PeptideProphet [10] probability (P) score of ≥0.9 from a total of 568 LC-MS/MS runs. The overall false discovery rate for spectrum assignment was calculated to be 1.2% based on PeptideProphet cutoffs. Of the 13,779 distinct peptides with P ≥0.9 encompassed in the analysis, 13,461 peptides were successfully mapped to the Ensembl Mouse [11] release 43, which was built on the NCBI m36 mouse assembly. Of the set of 13,779 distinct peptides, 9,170 (67%) were identified with at least 2 spectra. Cys-containing, Trp-containing, and Lys-containing peptides represented 3,709 (27%), 2,067 (15%), and 8,392 (61%) of the total peptides, respectively (Table 1).

**Table 1:** Summary of peptides identified with a PeptideProphet P score ≥0.9

Peptide	Counts	Percentage
Total assignment above threshold	800,507	
Total correct assignment	791,069	
Total incorrect assignment	9,438	
Spectrum assignment false discovery rate	0.012	
		
Total distinct peptides	13,779	100.00
Distinct peptides mapped to human genome	13,461	97.69
Possible proteins implicated in mapping	10,674	
Simple reduced proteins	4,084	
Simple reduced genes	3,580	
Unambiguously mapped proteins	1,590	
		
Total distinct peptides, presented in ≥2 samples	9,170	66.55
Total singleton distinct peptides	4,609	33.45
Cys-containing distinct peptides	3,709	26.92
Trp-containing distinct peptides	2,067	15.00
Lys-containing distinct peptides	8,392	60.90
		
Total distinct peptides from colon cancer versus normal	5,958	
Total distinct peptides from breast cancer versus normal	8,874	
Total distinct peptides from pancreatic cancer versus normal	7,753	
Total distinct peptides from ovarian cancer versus normal	5,368	
Total distinct peptides in all four reference sets	2,897	21.02
Total distinct peptides in three reference sets	1,534	11.13
Total distinct peptides in two reference sets	2,415	17.53

**Figure 1:**
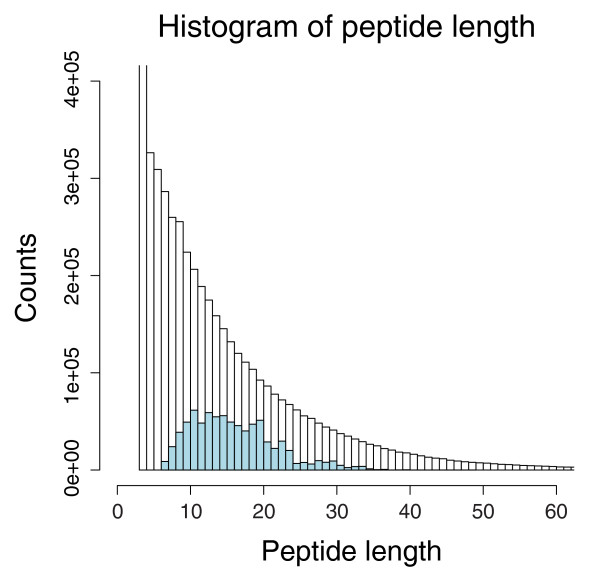
Characterization of distinct peptides identified from mouse plasma reference sets. The histogram of peptide length of unique sequences in mouse PeptideAtlas (blue) is overlaid on an *in silico *tryptic digest of the IPI mouse database (black).

The mean peptide length for the 13,779 peptide set was 16, with a range of 6-49 amino acids. There was a bias in the distribution of peptide length, which favored relatively long peptides (Figure 1). A similar finding from human proteome studies was previously reported [12]. The under-representation of short peptides may result from losses due to reduced sequence-specific fragment ions, or difficulty in distinguishing them from noise due to low m/z values. The mean molecular weight of this set was approximately 1,750 Da with a range of 640-4,100 Da. The majority of peptides identified were either neutral or acidic, with an average pI of approximately 6. There were 5958, 8874, 7753, and 5368 distinct peptides identified for the 4 reference sets (Table 1). The number of peptides identified may relate to variability in protein levels between reference sets, particularly for abundant proteins, which affects mass spectrometer peptide sampling and variability in protein recovery with sample processing.

### Identification of 2,982 proteins in mouse plasma using ProteinProphet

A combined search of data from all four reference sets was done using ProteinProphet [13]. This yielded 2,982 distinct International Protein Index (IPI) identifications corresponding to 2,631 known genes plus 281 hypothetical proteins with an error rate less than 5%. Among these, 2,131 (71%) proteins were identified with at least 3 unique peptides, 2,600 (87%) with at least 2 unique peptides, and 382 with only one unique peptide (singlets, 13%). Among these singlets, 140 (37%) were observed only once within the whole study, and thus are likely the major source of false identifications. Cytoplasmic proteins contributed the most, at approximately 29%; extracellular, nucleus, and plasma membrane proteins accounted for 17%, 17%, and 14%, respectively, based on ingenuity pathway analysis [14]. The limited contribution of secreted and surface membrane proteins to the overall total may be the result of release through non-secreted pathways and cell turnover. The tissue distribution and gene expression levels of this set of proteins was investigated based on the mouse SymAtlas (Novartis Research Foundation) [15]. The tissue with the maximum expression for a given gene was assigned to that particular gene. Approximately 8% of identified proteins had the highest expression of their corresponding genes represented by liver (mRNA per gram tissue), which is considered a major source of abundant plasma proteins. The range of MS/MS events corresponding to high confidence protein identifications based on two peptides or more varied between approximately 50,000 and 2. We previously observed a significant correlation between the number of MS/MS events for a given protein and its plasma protein concentration (log(MS/MS events) = (0.623 × log(Protein concentration)) + 0.0625) (unpublished data). We infer, therefore, that abundance of mouse plasma proteins identified in this study may span a logarithmic range of 7.

### Occurrence of cleaved protein forms

**Figure 2:**
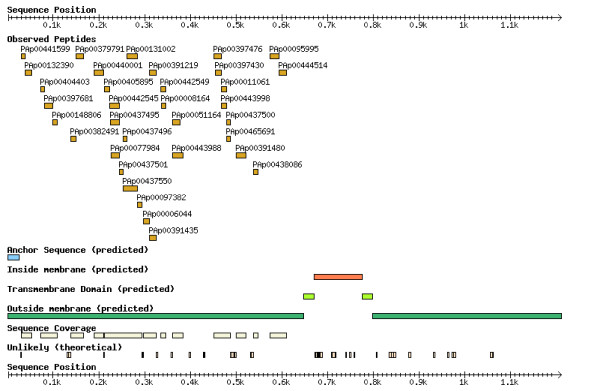
Peptide identification and distribution of mouse EGFR. A total of 34 distinct peptides were identified from approximately 4,000 MS2 events. All peptides are from the extracellular domain.

Proteins undergo numerous post-translational modifications, notably cleavages in the case of proteins shed into the circulation. The mouse PeptideAtlas may be queried to determine the distribution of peptides identified for a given protein and the occurrence of cleaved forms in particular fractions. This is illustrated for the epidermal growth factor receptor (EGFR), which is a single-pass type I membrane protein [16], as an example of the depth of analysis achieved and of the capabilities of the mouse PeptideAtlas. Detection of over-expressed EGFR is of relevance to a number of disease processes [17]. The trans-membrane region is located at amino acids 646-668, and the extracellular domain is between amino acids 25-645 (Figure 2). A total of over 4,000 MS2 events in mouse PeptideAtlas corresponding to EGFR matched 34 distinct peptides that spanned exclusively the extracellular domain of the protein, resulting from cleavage and release of this domain. The PeptideAtlas provides a graphic interface for protein fragmentation and can be used as a tool for comparisons between different species. Of interest, one peptide derived from mouse EGFR, PAp00148806 (IPLENLQIIR amino acids 99-108, lab 34), was also identified in the PeptideAtlas analysis of the Human Proteome Organization (HUPO) human plasma samples as the mouse and the human share the same sequence for this peptide [9].

### Observed splice isoforms

Approximately 3,000 distinct peptides were identified that spanned exon boundaries, of which 1,717 were observed at least twice. Among these, 180 peptides were observed in all 4 mouse plasma pools/experiments, and mapped to proteins with a unique genome location. The database represents a useful resource for validation of splice isoforms predicted in the Ensembl mouse genome. For example, PAp00024736, with a peptide sequence DQGSCGSCWAFGAVEAISDR, was mapped to a single protein cathepsin B precursor with a unique genome location on mouse chromosome 14. This peptide was observed a total of 39 times in multiple fractions and in all 4 experiments. The first nine amino acids (DQGSCGSCW) covered one exon from genome location 62089806 to 62089832, while the rest (AFGAVEAISDR) were located on another exon at genome location 62090517 to 62090549. Cathepsin B regulates the hydrolysis of proteins with broad specificity for peptide bonds [18].

A list of approximately 950 peptides that did not map to any annotated gene in the mouse genome was developed, with an overall 1.7% false discovery rate for peptide identification. These represent putative novel open reading frames. The majority (61%) had at least two observations. One example is PAp00438183, with a sequence of RPQMVEGDHGDEIFSVFGAPLLK, which was identified over 300 times and in all 4 experiments. The peptide was mapped to a single protein, IPI00138342, the liver carboxylesterase N precursor. Its sequence matched to the coding region of this protein on chromosome 8 with 91% identity.

### Identification of novel alternative splice isoforms

Alternative splicing plays a major role in protein diversity without significantly increasing genome size. Aberrations in alternative splice variants are known to contribute to a number of diseases [19]. As highlighted recently in the Encode project [20], the extent of transcript structural variation in mammalian genomes has been under-appreciated. To identify novel splice isoforms that are translated into protein products, we interrogated the intact protein analysis system (IPAS; see Materials and methods) data sets using a protein sequence collection containing the products of both known and hypothetical transcripts.

A target database with over 10 million sequences was built upon the ECgene [21] and Ensembl mouse [11] databases as described in Materials and methods and in Fermin *et al*. [22]. An extensive computational analysis for a reference set was done to identify novel forms. Using a X!Tandem expect value of <1e-3 as a threshold, we identified a total of 12,461 proteins and 8,154 distinct peptides matching 147,051 spectra in the target database. Among these, 7,291 distinct peptides (90%) were in multi-peptide sets and 863 (10%) in single peptide identification sets. At this threshold, 81 distinct reversed sequence proteins (0.65%) and 53 reversed sequence peptides (0.65%) matched 69 distinct spectra.

The splice isoforms derived from a gene share exons with each other. Further, specific peptides may occur in several members of a paralogous family of proteins. To obtain a measure of the number of independent protein identifications, it is necessary to integrate protein identifications into covering sets (see Materials and methods). To be included in the integrated protein list, peptide spectral matches need to be unique and not explained by another protein in the list. The integrated list for the set of protein identifications including novel splice isoform translation products contained a total of 1,324 distinct proteins. Multiple splice isoforms were identified for a number of proteins that have been suggested as potential disease biomarkers in previous studies: Cpn1 (carboxypeptidase N, polypeptide 1), Pzp (pregnancy zone protein), Fabp5 (fatty acid binding protein 5) and Mbl2 (mannose binding lectin) [23]. In selecting proteins to be members of the covering sets, our algorithm gives preference to annotated protein sequences. Of the integrated 1,324 protein sets, 1058 (80%) were annotated gene products (that is, proteins in the Ensembl protein collection) and 199 (15%) were found only in the ECGene collection of novel transcripts. Note that in many cases, the set of identifications 'covered' by an annotated protein will also contain proteins derived from previously un-annotated transcripts.

### Comparative analysis of mouse and human splice isoforms

**Figure 3:**
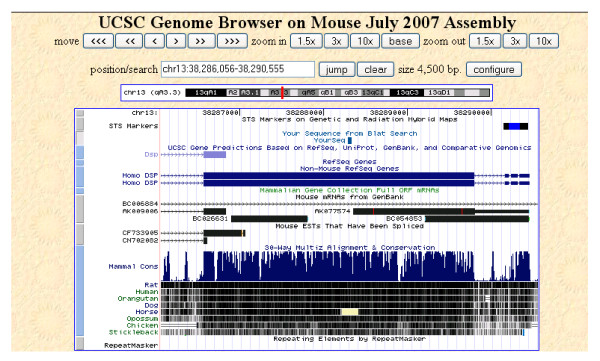
Genomic mapping of mouse peptide LLEAQIATGGIIDPK using UCSC Blast program. Mouse splice variant M13C2563_1_s386_e8960_1_rf2_c1_n0 was identified with 13 distinct peptides. Twelve peptides matched a predicted mouse protein similar to desmoplakin using a homolog search against the NCBI NR database. However, all 13 peptides were homologous to the human desmoplakin sequence. Of note, mouse peptide LLEAQIATGGIIDPK aligns to the coding sequence of human desmoplakin, but not to the annotated mouse desmoplakin gene.

We examined mouse splice variants identified as homologues of human counterparts. Similarities were uncovered with human peptides. A case in point is mouse splice variant M13C2563_1_s386_e8960_1_rf2_c1_n0, which was identified with 13 distinct peptides from 19 spectra through the genomic database search. Peptides LLEAQIATGGIIDPK, GFFDPNTEENLTYLQLK, and LNDSILQATEQR were identified by four, three and two spectra, respectively. However, when all 13 peptides were searched against the NCBI NR database using the blastp program, 12 peptides matched to a predicted mouse protein similar to desmoplakin protein. In contrast, all 13 peptides were found to be homologous to the human desmoplakin sequence. The alignment of peptide LLEAQIATGGIIDPK using the UCSC Blast program is shown in Figure 3. This peptide aligns to the coding sequence of human desmoplakin, but not to the annotated mouse desmoplakin gene. Therefore, our data clarify similarities between the mouse and the human coding sequences for this gene. Such detailed analysis of splice variants may identify novel alternative splicing relevant to disease.

### Protein fractionation as a means to characterize splice isoform products

**Figure 4:**
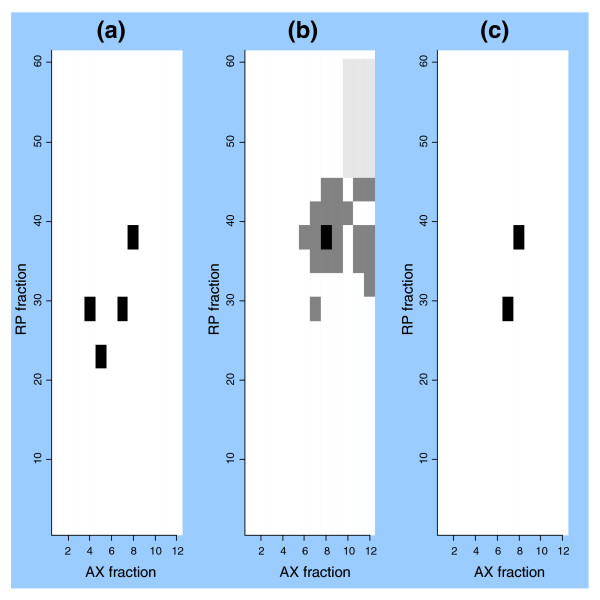
IPAS mapping of splice isoform variants for the MHC H2 K1 K gene. Intact proteins were subjected to anion exchange (AX) and reverse phase (RP) chromatography fractionation. Each fraction was then subjected to tryptic digestion and LC-MS/MS analysis. Peptides derived from novel exons falling within the MHC 2 K1 K gene were identified. Spectral counts for matching peptides derived from this gene are plotted as a function of AX and RP fractionation. The color of each cell indicates the number of matching spectra: black = high; gray = medium; light gray = low; white = zero. **(a) **The distribution of spectra matching the major annotated splice isoform (ENSMUST00000025181). **(b) **The distribution of spectra matching the novel splice isoform (M17C4317_13_s2_e917_1_rf2_c1_n0|). **(c) **The distribution of spectra matching peptides found in both the major annotated splice isoform for this gene (ENSMUST00000025181) and the novel splice isoform (M17C4317_13_s2_e917_1_rf2_c1_n0|).

When the protein products of alternative splice isoforms differ in structure, we expect that the physical properties of these proteins, in particular their fraction location, may vary. Finding evidence for non-neighboring fractions with distinct peptide content for the same protein supports the identification of multiple products from the same gene. Figure 4 illustrates this analysis for the major histocompatibility complex (MHC) H2 K1 K region antigen. Intact proteins were subjected to anion exchange (AX) and reverse phase (RP) chromatography fractionation in this study. Each fraction was then subjected to tryptic digestion and LC-MS/MS analysis. Figure 4 shows the distribution of peptides derived from this gene among the AX and RP chromatography fractions. The unique peptides identified in the major splice isoform and alternative splice variants were found in separate fractions, consistent with translation of the different splice isoforms yielding distinct protein products with distinct physical properties. The MHC H2 K1 K region gene encodes one of the MHC class 1 antigens, which may be altered in tumors [24].

### Pathway annotation of identified mouse plasma proteins

To determine whether sufficient depth of analysis was achieved to identify proteins that are relevant to disease, we performed ingenuity pathway analysis [14] for 1,058 proteins that were fully annotated to known proteins. A number of known genes may be candidate disease biomarkers based on splice variants. For example, CD44 [25] is a cell-surface glycoprotein that participates in a variety of cellular functions, including tumor metastasis. Alternative splicing generates a diverse collection of structurally and functionally distinct protein products from this gene [14,26,27]. CD44 gene products, consisting of three splice isoforms, are found in the integrated set of proteins defined by the lead peptide YGFIEGNVVIPR. This group of 3 splice isoforms was identified with 3 distinct peptides from 14 spectra in our data set. All members of this group included all three peptides.

The extracellular matrix protein 1 is another example where the major splice isoform and two novel alternative splice isoforms were found in the integrated list. Extracellular matrix protein 1 is a secreted protein that has been implicated in cell proliferation, angiogenesis and differentiation. This protein is preferentially expressed in epithelial tumors and has been suggested as a potential cancer biomarker [28].

## Discussion

Our study has yielded an in-depth analysis of mouse plasma resulting in a high quality peptide database built from four reference pools. The mouse plasma PeptideAtlas currently contains more than 800,000 spectra corresponding to 13,000 peptides from approximately 3,000 proteins. Additions are expected from accumulation of new data sets resulting from other instrument platforms or sample processing protocols, other database search algorithms and analysis of disease models.

The identified proteins span an estimated 7 logarithm range of abundance encompassing prostate specific antigen, estimated to occur in plasma at approximately 1 ng/ml [29], to abundant plasma proteins present at 40 mg/ml. The ability to detect less abundant proteins is highly relevant to biomarker discovery as most known disease biomarkers occur at low-abundance in the range of prostate specific antigen. Detection of low abundance proteins results from a combination of extensive fractionation of intact proteins using an orthogonal two-dimensional system of AX and RP chromatography, and high resolution LC-MS/MS analysis. The methodology also allows detection of protein cleavage products and splice isoforms.

Some similarities and some differences between human and mouse plasma have emerged from our analysis. An extensive pattern of cleavage of biological significance has been reported previously for human complement factor C3 [30], and our data demonstrate similar findings for the mouse. In contrast, for EGFR, a marked difference in abundance based on number of spectra was observed between human (10 spectra) and mouse (>4,000), which may be related to differences in sources of EGFR between mouse and human based on gene expression analysis [15]. EGFR has the highest expression in liver in mouse, which is not the case for humans. Alternative splicing is an important source of protein diversity [31,32], and deep sampling of the plasma proteome as accomplished in this study is relevant to the identification of novel splice isoforms. Variation in the delicately controlled process of splicing may occur in disease states. Alternative splicing in cancer has been investigated on a small scale [25]. Proteomic data as obtained in this study provide a complementary approach to annotate the genome [33,34], and represent a useful resource for the discovery of alternatively spliced forms. We identified novel splice isoforms of known genes as well as previously un-annotated open reading frames [22]. An assessment of the relevance of identified splice isoforms as disease biomarkers would be of interest.

## Conclusion

We have developed the mouse plasma PeptideAtlas database, along with its web interface, as a means for depositing mass spectrometry derived protein and peptide identifications in mouse plasma. This initial release contains data derived from 568 LC-MS/MS runs of plasma fractions. A total of 13,779 distinct peptides have been identified with high confidence and are included in this release, which can be searched and downloaded. An important component comprises novel isoforms and transcript variants not previously predicted from genome analysis that have been identified.

## Materials and methods

### Sample preparation and mass spectrometry analysis

The data for the mouse plasma PeptideAtlas was produced from an initial set of four reference plasma samples. Each represented a mixture of plasma from a mouse model of cancer and its matched control as presented in Table 2. All experiments were carried out using the IPAS methodology [29]. Pooled plasma samples, immunodepleted of the abundant proteins albumin, IgG, and transferrin using an Ms-3 column (Agilent, Wilmington, DE, USA) were subjected to an extensive two-dimensional fractionation schema using AX chromatography followed by RP chromatography, yielding approximately 150 fractions per plasma pool. Each fraction was subjected to in-solution digestion with trypsin followed by LC-MS/MS analysis on either an LTQ-FT or LTQ-Orbitrap mass spectrometer (Thermo-Finnigan, Waltham, MA, USA) coupled to a nanoAcquity nanoflow chromatography system (Waters, Milford, MA, USA).

**Table 2:** Sources of plasma reference sets

Sample tag	Sample ID	Mouse model	Strain	Number of fractions analyzed	Instrument
Pancreatic cancer and matched control	347	[42] Kras and INK/ARF	FVB/n	163	Thermo LTQ-FT
Breast cancer and matched control	348	[43] Her2/Neu	FVB	144	Thermo Orbitrap
Ovarian cancer and matched control	349	[44] *LSL-K-ras*^G12D/+ ^*Pten*^loxP/loxP^	LSL	144	Thermo LTQ-FT
Colon cancer and matched control	350	[45] D580	129/B6	117	Thermo LTQ-FT

Each set consisted of a pool of plasma from a mouse model of cancer and its matched wild-type control.

### Database searches and building the mouse plasma PeptideAtlas

Acquired data were automatically processed through the Computational Proteomics Analysis System [35] pipeline. The database search was initiated using the X!Tandem algorithm [12] with the comet k-score module plug-in [36]. The mouse IPI database [37], version 3.13, was used throughout the project. All results of sequence searching were subsequently processed through PeptideProphet [10] for peptide validation and quality control, using the Trans Proteomic Pipeline [38], version 2.9.9. All identifications with a PeptideProphet probability (P) 0.9 and above were combined to form a master list of observed peptides across all four reference sets. All experimental data were loaded into a proteomics analysis database module under the Systems Biology Experiment Analysis Management System [39]. The identified peptide sequences were then mapped to the Ensembl mouse release 43 proteome based on NCBI mouse genome build 36, and the results were loaded into the PeptideAtlas relational database [40,41].

ProteinProphet [13] was run on these reference data sets in a combined mode for protein identification. Proteins with less than 5% error rate from ProteinProphet were further characterized with respect to tissue expression. Mouse GNF 1M GeneAtlas, developed at the Genomics Institute of the Novartis Research Foundation [15], was downloaded, and the tissue with the maximum expression for a given gene was assigned to that particular gene/protein. Subcellular location and major functional network categorizations were based on ingenuity pathway analysis [14].

### Analysis of alternative splice isoforms

A target database, which had a total of 10,381,156 protein sequences, was generated [22] as follows: cDNA sequences from the ECgene database [21] and from the Ensembl mouse database [11] were obtained in FASTA format; each sequence set was translated separately in 3 reading frames and the first instance of every protein sequence longer than 14 amino acids was recorded; the sequences were combined and then filtered for redundancy (preference was given to protein sequences originating from an Ensembl transcript); and a collection of common protein contaminants and reverse protein sequences was appended to the sequence database. The formatted peak list files from each dataset were searched against the target database using X!Tandem; peptides identified with an X!Tandem expect value of <0.001 were extracted for further analyses.

### Protein peptide identifications to determine minimal covering sets

Many transcription units produce transcripts with multiple splice isoforms that share exons. This often precludes assigning a peptide as the translation product of a specific splice isoform. To avoid redundancy in analysis, protein identifications were integrated to determine the minimum number of covering sets. A covering set is defined as a set of proteins containing all of the identified peptides. The minimal covering set is the covering set with the smallest number of members.

Defining a minimal covering set is a nondeterministic polynomial-time hard (NP hard) calculation, so exact definition of the minimal covering set is not feasible for data sets of the size found in protein/peptide identifications. Instead, we employ a heuristic approach to find approximately optimal solutions. Each member of the covering set of proteins is defined by a 'lead peptide'. The minimal covering set of proteins needed to explain the observed peptide identification data was generated as follows: step 1, a list of peptides identified in all experiments was created; step 2, the peptide list was ordered by the number of spectra matching the peptide; step 3, the peptide matching the largest number of spectra was selected (the 'lead peptide'); step 4, a list of all proteins containing this peptide was generated; step 5, the protein list was sorted by the number of spectra matching peptides derived from the protein; step 6, the Ensembl protein having the largest number of matching spectra was selected (in the event of ties, the protein having the largest number of distinct peptides was selected; if there were no Ensembl proteins in the list, the splice variant with the largest number of spectra and distinct peptides was selected); step 7, all peptides contained in this protein were removed from the peptide list; step 8, the procedure was repeated beginning with step 3 until no unassigned peptides remained, thus generating a covering set of proteins that contained all of the identified peptides. Each protein in the covering set contains at least one peptide that is unique to this protein among all of the members of the covering set.

## Abbreviations

AX, anion exchange; EGFR, epidermal growth factor receptor; IPAS, intact protein analysis system; IPI, International Protein Index; LC-MS/MS, liquid chromatography-tandem mass spectrometry; MHC, histocompatibility complex; P, probability; RP, reverse phase.

## Authors' contributions

QZ designed the study, performed data analysis, and drafted the manuscript. RM carried out the genomic data analysis and helped with the draft. EWD carried out the building of the mouse plasma PeptideAtlas and made it public. SJP, VMF, HW, and LFN performed the IPAS experiments. RAD, NB, DD, KEH, RK, TJ, and KP provided samples. RA, GSO, and DJS were involved in data analysis and helped with the draft. SMH designed the study and revised the draft. All authors read and approved the final manuscript.
